# Structure cristalline de la triple molybdate Ag_0.90_Al_1.06_Co_2.94_(MoO_4_)_5_

**DOI:** 10.1107/S2056989015005290

**Published:** 2015-03-21

**Authors:** Rawia Nasri, Saïda Fatma Chérif, Mohamed Faouzi Zid

**Affiliations:** a Laboratoire de Matériaux et Cristallochimie, Faculté des Sciences de Tunis, Université de Tunis El Manar, 2092 El Manar Tunis, Tunisia

**Keywords:** triple molybdates, hexa­kis(molyb­date), open-framework structure, crystal structure

## Abstract

Silver(I) aluminiun tricobalt(II) penta­kis­[tetra­oxidomolybdate(VI)], Ag_0.90_Al_1.06_Co_2.94_(MoO_4_)_5_, was synthesized using a solid-state reaction at 845 K. The structure can be described as a three-dimensional framework formed from dimeric *M*_2_O_10_ (*M* = Co/Al) and trimeric *M*_3_O_14_ units linked to MoO_4_ tetra­hedra by sharing corners, with the cavities occupied by disordered Ag^+^ cations. It is shown that the Co and Al atoms occupy common positions with different occupancies. The Ag^+^ cations are located at two different sites with occupancies of 0.486 (1) and 0.408 (1). The title coumpond is isotypic with NaMg_3_Al(MoO_4_)_5_ and NaFe_4_(MoO_4_)_5_. Differences and similarities with other related structures are discussed.

## Abstract 2

A new triple hexa­kis­(molybdate), Ag_0.90_Al_1.06_Co_2.94_(MoO_4_)_5_, was synthesized using a solid-state reaction at 845 K. Dimers *M*_2_O_10_ (*M* = Co/Al) and trimers *M*_3_O_14_ link to the MoO_4_ tetra­hedra by sharing corners and form a three-dimensional framework with the inter­stitial sites occupied by Ag^+^ cations.

## Contexte chimique   

Les molybdates triples des métaux de transition ont un champ prometteur pour diverses applications: catalyse (Ivanov *et al.*, 1998 ▸), spectroscopie (Méndez-Blas *et al.*, 2004 ▸). L’assemblage octa­èdres-tétraèdres dans ces matériaux conduit à des charpentes ouvertes présentant des propriétés physiques importantes, en particulier la conduction ionique (Judeinstein *et al.*, 1994 ▸; Sanz *et al.*, 1999 ▸). L’exploration du système *A*–Co–Al–Mo–O (*A* = ion monovalent) nous a permis d’élaborer un nouveau matériau de formulation Ag_0.90_Al_1.06_Co_2.94_(MoO_4_)_5_. Un examen bibliographique montre que le matériau étudié est isostructural aux composés: NaMg_3_Al(MoO_4_)_5_ (Hermanowicz *et al.*, 2006 ▸) et NaFe_4_(MoO_4_)_5_ (Muessig *et al.*, 2003 ▸).

## Commentaire structurelle   

**Figure 1:**
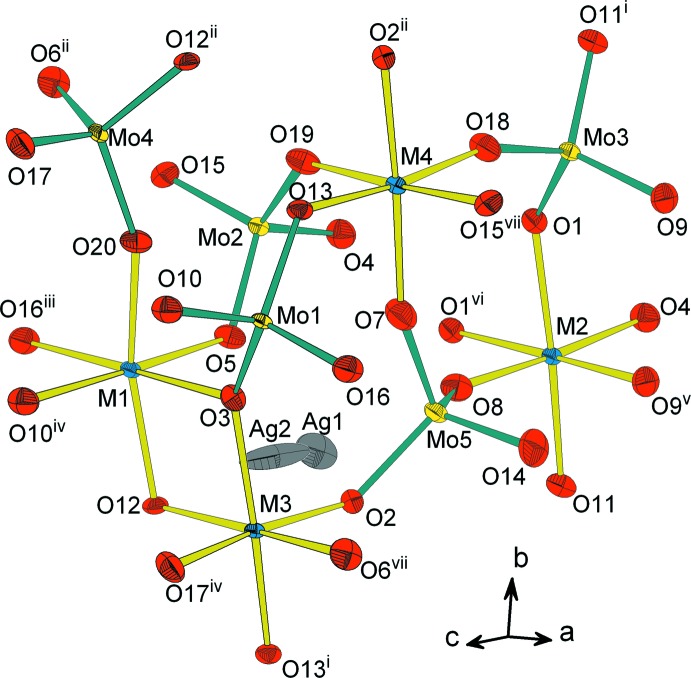
Représentation des polyèdres de coordination de l’unité structurale dans Ag_0.90_Al_1.06_Co_2.94_(MoO_4_)_5_. Les éllipsoïdes ont été définis avec 50% de probabilité. [Code de symétrie: (i) *x*, *y* + 1, *z*; (ii) *x*, *y* − 1, *z*; (iii) *x* + 1, *y*, *z*; (iv) −*x* + 1, −*y* + 1, −*z*; (v) −*x* + 2, −*y* + 1, −*z* + 1; (vi) −*x* + 1, −*y* + 1, −*z* + 1; (vii) *x* − 1, *y*, *z;* M = Co/Al.]

**Figure 2:**
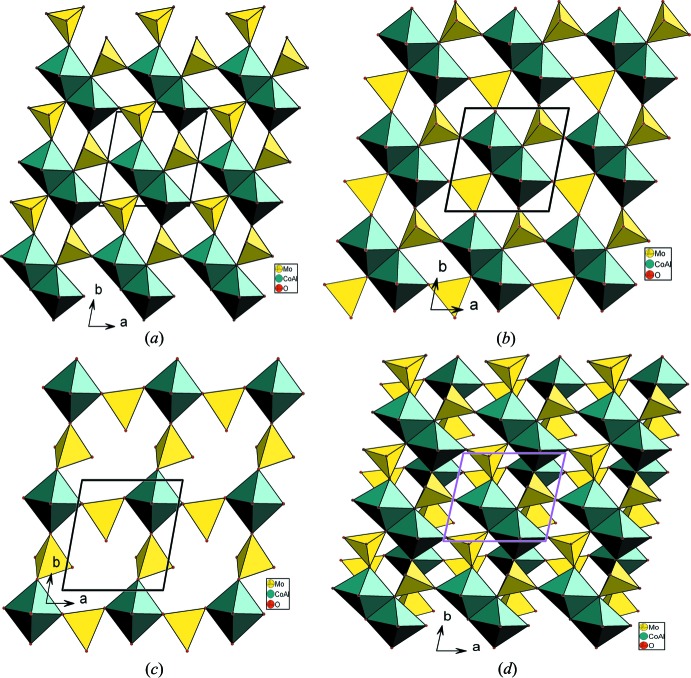
Projection: (*a*) d’une couche de type *A* disposée parallèlement au plan (001), (*b*) d’une couche de type *D* dans le plan (001), (*c*) d’une couche de type *C* dans le plan (001), (*d*) d’une bicouche dans le plan (001).

L’unité structurale renferme un dimère *M*_2_O_10_ (*M* = Co/Al), deux octa­èdres *M*O_6_ et cinq tétraèdres MoO_4_ reliés par mise en commun de sommets. La compensation de charges dans la structure est assurée par les cations Ag^+^ (Fig. 1 ▸). La charpente anionique peut être décrite moyennant la succession de différents types de couches reliées par partage de sommets et d’arêtes. Elle peut être subdivisée en couches de type *A*, *B*, *C* et *D*. Les couches de type *A* sont formées par les dimères *M*_2_O_10_ (*M* = Co2/Al2) reliés par mise en commun de sommets uniquement avec les tétraèdres Mo3O_4_ disposés en ‘*trans’* (Fig. 2 ▸ *a*). Dans les couches de type *D*, les dimères *M*_2_O_10_ (*M* = Co1/Al1, Co3/Al3), se connectent par mise en commun de sommets avec les tétraèdres Mo1O_4_ et Mo4O_4_, dans lequels les sommets non engagés dans la couche sont tous orientés selon la même direction en ‘*cis’* (Fig. 2 ▸ *b*). Dans les couches de type *C*, les octa­èdres *M*O_6_ (*M* = Co4/Al4) et les tétraèdres Mo2O_4_ et Mo5O_4_ se connectent par mise en commun de sommets pour former des chaînes classiques de type *M*MoO_8_ (Fig. 2 ▸ *c*).

**Figure 3:**
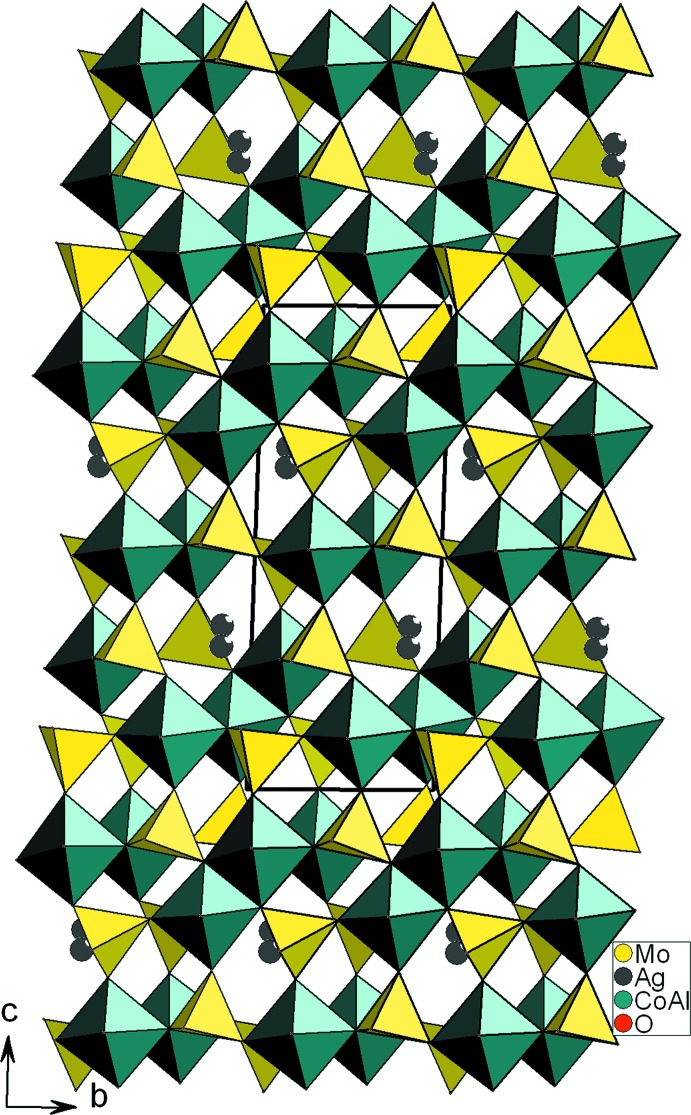
Projection de la structure de Ag_0.90_Al_1.06_Co_2.94_(MoO_4_)_5_, selon *a*.

La disposition particulière, des tétraèdres MoO_4_ en ‘*cis’* dans les couches de type *D* et en ‘*trans*’ dans les couches *A*, respectivement permet la jonction de ces dernières par ponts mixtes pour conduire à des doubles couches de type *B* (Fig. 2 ▸ *d*). La jonction des différentes couches *A* et bicouches *B* parallèlement au plan (001), selon la disposition *A*–*BB*–*A*–*BB* par ponts mixtes de type *M*–O–Mo conduit à une charpente tridimentionnelle possédant des canaux dans lesquels résident les cations Ag^+^, mais excentrés (Fig. 3 ▸).

Un examen des caractéristiques géométriques relevées de l’étude structurale montre que les distances moyennes dans les tétraèdres MoO_4_ et dans les octa­èdres *M*O_6_ (*M* = Co/Al), sont égales respectivement à 1.762 (4) et 2.036 (4) Å. La première Mo—O, est conforme à celles rencontrées dans la litérature (Ennajeh *et al.*, 2013 ▸; Engel *et al.*, 2009 ▸; Huyghe *et al.*, 1991 ▸). La seconde *M*—O (*M*=Co/Al), s’avère une moyenne entre celles Co^II^–O (Engel *et al.*, 2009 ▸; Sanz *et al.*, 1999 ▸) et Al—O (Brik & Avram, 2011 ▸; Hermanowicz *et al.*, 2006 ▸). Dans les dimères *M*_2_O_10_, la distance courte métal–métal égale à 3.109 (8) Å, pourrait conduire à des propriétés magnétiques (Feng *et al.*, 1997 ▸). De plus, le calcul des charges des ions, utilisant la formule empirique de Brown & Altermatt (1985 ▸), conduit aux valeurs des charges des ions suivants: Mo1 (6.047), Mo2 (6.012), Mo3 (5.949), Mo4 (5.965), Mo5 (5.993), (Co1/Al1) (2.140), (Co2/Al2) (2.314), (Co3/Al3) (2.312), (Co4/Al4) (2.481), Ag1 (0.965) et Ag2 (0.998). En effet, en tenant compte des taux d’occupation des sites, la charge globale calculée des cations restants [+10,1(2)] est égale en module à celle de l’ion molybdate [Mo_5_O_20_]^10−^.

Un examen rigoureux des travaux antérieurs montre une analogie structurale entre les connections des polyèdres dans les composés appartenant à la famille de type alluaudite Na_3_In_2_As_3_O_12_ et Na_3_In_2_P_3_O_12_ (Lii & Ye, 1997 ▸), le matériau Ag_2_Co_2_(MoO_4_)_3_ (Tsyrenova *et al.*, 2004 ▸) et les différentes variétés du composé K_2_Co_2_(MoO_4_)_3_ (Engel *et al.*, 2009 ▸). Dans ces phases, une différence nette a été observée dans les charpentes anioniques. En effet, on remarque que dans le cas des alluaudites, les dimères adoptent une disposition perpendiculaire les uns aux autres. Contrairement à notre structure dans laquelle les dimères sont disposès d’une façon parallèle.

**Figure 4:**
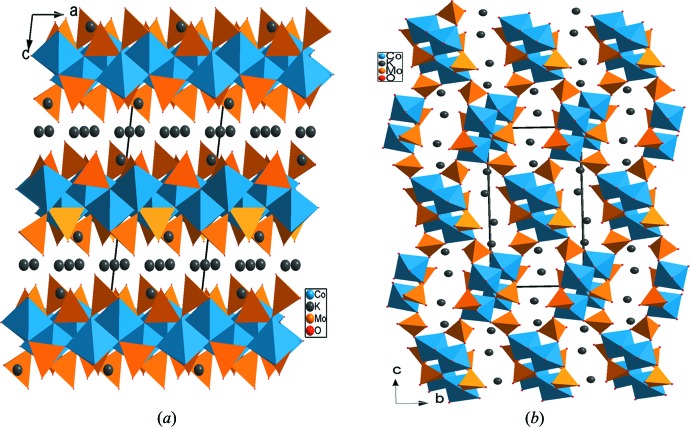
Projection de la structure de: (*a*) *β*-K_2_Co_2_(MoO_4_)_3_, selon *b*, (*b*) *α*-K_2_Co_2_(MoO_4_)_3_, selon *a*.

La charpente anionique dans le composé K_2_Co_2_(MoO_4_)_3_ présente contrairement à notre structure des tétramères au lieu des dimères et trimères. L’association, par partage de sommets, des tétramères avec les tétraèdres MoO_4_ conduit dans la forme *β*-K_2_Co_2_(MoO_4_)_3_ à une structure en couches (two-dimensional) (Fig. 4 ▸ *a*). Par contre, leur jonction dans la forme *α*-K_2_Co_2_(MoO_4_)_3_ engendre une charpente tridimensionnelle possédant des canaux allongés où résident des cations potassium (Fig. 4 ▸ *b*).

## Synthèse et cristallisation   

Dans le but de préparer un composé de formulation analogue à NaMg_3_Al(MoO_4_)_5_ ayant des propriétés physiques intéressantes, nous avons voulu synthétiser la phase AgAlCo_3_(MoO_4_)_5_. Un mélange de réactifs: AgNO_3_ (Merck, 101510), Co(NO_3_)_2_·6H_2_O (FLUKA, 60832), Al_2_O_3_ (FLUKA, 60109) et (NH_4_)_2_Mo_4_O_13_ (FLUKA, 69858) a été pris dans les proportions tel que les rapports sont Ag:Al:Co:Mo=1:1:3:5. Après un broyage poussé dans un mortier en agate, le mélange a été mis dans un creuset en porcelaine préchauffé à l’air à 673 K pendant 12 heures en vue d’éliminer les composés volatils. Il est ensuite porté jusqu’à une température de synthèse proche de celle de la fusion à 845 K. Le mélange est abandonné à cette température pendant deux semaines pour favoriser la germination et la croissance des cristaux. Par la suite, il a subi en premier lieu un refroidissement lent (5°/jour) jusqu’à 800 K puis rapide (50°/h) jusqu’à la température ambiante. Des cristaux de couleur rouge, de taille suffisante pour les mesures des intensités, ont été séparés du flux par l’eau chaude. Une analyse qualitative au MEB de marque FEI et de type *QUANTA* 200 confirme la présence des éléments chimiques attendus: Ag, Al, Co, Mo et l’oxygène.

## Affinement   

Détails de donnés crystallines, collection de donnés et affinement sont résumés dans le tableau 1 ▸. La structure a été résolue par des méthodes directes de *SHELXS97* (Sheldrick, 2008 ▸), et inter­pretée en partant de la formule AgAlCo_3_Mo_5_O_20_ similaire au composé isotype NaAlMg_3_Mo_5_O_20_. Un examen de la carte de Fourier différence montre des résidus non négligeables autour des cations Co^2+^ et Ag^+^. L’affinement, en se basant sur les grandeurs géométriques, a été mené d’une part avec des taux d’occupation variables pour les atomes de cobalt et de l’aluminium occupant statiquement les mêmes positions et ayant les mêmes ellipsoïdes utilisant les deux fonctions EXYZ et EADP autorisées par le programme *SHELXL97* (Sheldrick, 2008 ▸), et d’autre part en considérant que l’ion Ag^+^ est reparti sur deux positions proches dans la structure. En effet, l’affinement de tous les paramètres variables conduit à des ellipsoïdes bien définis. Les densités électroniques maximum et minimum restantes dans la carte de Fourier différence sont acceptables et sont situées respectivements à 0.92 Å de Mo4 et à 0.93 Å de Mo3.

## Supplementary Material

Crystal structure: contains datablock(s) I Crystal structure: contains datablock(s) I. DOI: 10.1107/S2056989015005290/vn2089sup1.cif

Crystal structure: contains datablock(s) I Crystal structure: contains datablock(s) I. DOI: 10.1107/S2056989015005290/vn2089sup1.cif

Structure factors: contains datablock(s) I. DOI: 10.1107/S2056989015005290/vn2089Isup2.hkl

CCDC reference: 1054352

Additional supporting information:  crystallographic information; 3D view; checkCIF report

## Figures and Tables

**Table 1:** Dtails exprimentaux

Donnes crystallines	
Formule chimique	Ag_0,90_Al_1,06_Co_2,94_(MoO_4_)_5_
*M*_r_	1098,64
Systme cristallin, groupe d’espace	Triclinique, *P* 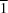
Temprature (K)	298
*a*, *b*, *c* ()	6,8547(8), 6,9410(8), 17,597(2)
, , ()	87,958(6), 87,462(6), 78,818(4)
*V* (^3^)	820,20(16)
*Z*	2
Type de rayonnement	Mo *K*
(mm^1^)	7,79
Taille des cristaux (mm)	0,22 0,16 0,12

Collection de donnes	
Diffractomtre	EnrafNonius CAD-4
Correction d’absorption	scan (North *et al.*, 1968 ▸)
*T*_min_, *T*_max_	0,233, 0,407
Nombre de rflexions mesures, indpendantes et observes [*I* > 2(*I*)]	3912, 3527, 3086
*R*_int_	0,029
(sin /)_max_ (^1^)	0,638

Affinement	
*R*[*F* ^2^ > 2(*F* ^2^)], *wR*(*F* ^2^), *S*	0,027, 0,070, 1,09
Nombre de rflexions	3527
Nombre de paramtres	287
_max_, _min_ (e ^3^)	1,28, 0,77

Programmes informatiques: *CAD-4 EXPRESS* (Duisenberg, 1992 ▸; Macek Yordanov, 1992 ▸), *XCAD4* (Harms Wocadlo, 1995 ▸), *SHELXS97* et *SHELXL97* (Sheldrick, 2008 ▸), *DIAMOND* (Brandenburg Putz, 2001 ▸) et *WinGX* (Farrugia, 2012 ▸).

## supplementary crystallographic information

### Crystal data

**Table:**

Ag_0.90_Al_1.06_Co_2.94_(MoO_4_)_5_	*Z* = 2
*M**_r_* = 1098.64	*F*(000) = 1010.92
Triclinic, *P*1	*D*_x_ = 4.448 Mg m^−^^3^
Hall symbol: -P 1	Mo *K*α radiation, λ = 0.71073 Å
*a* = 6.8547 (8) Å	Cell parameters from 25 reflections
*b* = 6.9410 (8) Å	θ = 10–15°
*c* = 17.597 (2) Å	µ = 7.79 mm^−^^1^
α = 87.958 (6)°	*T* = 298 K
β = 87.462 (6)°	Prism, red
γ = 78.818 (4)°	0.22 × 0.16 × 0.12 mm
*V* = 820.20 (16) Å^3^	

### Data collection

**Table:**

Enraf–Nonius CAD-4 diffractometer	3086 reflections with *I* > 2σ(*I*)
Radiation source: fine-focus sealed tube	*R*_int_ = 0.029
Graphite monochromator	θ_max_ = 27.0°, θ_min_ = 2.3°
ω/2θ scans	*h* = −8→1
Absorption correction: ψ scan (North *et al.*, 1968)	*k* = −8→8
*T*_min_ = 0.233, *T*_max_ = 0.407	*l* = −22→22
3912 measured reflections	2 standard reflections every 120 min
3527 independent reflections	intensity decay: 1.2%

### Refinement

**Table:**

Refinement on *F*^2^	Primary atom site location: structure-invariant direct methods
Least-squares matrix: full	Secondary atom site location: difference Fourier map
*R*[*F*^2^ > 2σ(*F*^2^)] = 0.027	*w* = 1/[σ^2^(*F*_o_^2^) + (0.0285*P*)^2^ + 3.7871*P*] where *P* = (*F*_o_^2^ + 2*F*_c_^2^)/3
*wR*(*F*^2^) = 0.070	(Δ/σ)_max_ = 0.001
*S* = 1.09	Δρ_max_ = 1.28 e Å^−^^3^
3527 reflections	Δρ_min_ = −0.77 e Å^−^^3^
287 parameters	Extinction correction: *SHELXL97* (Sheldrick, 2008), Fc^*^=kFc[1+0.001xFc^2^λ^3^/sin(2θ)]^-1/4^
0 restraints	Extinction coefficient: 0.00088 (12)

### Special details

**Table:**

Geometry. All e.s.d.'s (except the e.s.d. in the dihedral angle between two l.s. planes) are estimated using the full covariance matrix. The cell e.s.d.'s are taken into account individually in the estimation of e.s.d.'s in distances, angles and torsion angles; correlations between e.s.d.'s in cell parameters are only used when they are defined by crystal symmetry. An approximate (isotropic) treatment of cell e.s.d.'s is used for estimating e.s.d.'s involving l.s. planes.
Refinement. Refinement of *F*^2^ against ALL reflections. The weighted *R*-factor *wR* and goodness of fit *S* are based on *F*^2^, conventional *R*-factors *R* are based on *F*, with *F* set to zero for negative *F*^2^. The threshold expression of *F*^2^ > σ(*F*^2^) is used only for calculating *R*-factors(gt) *etc*. and is not relevant to the choice of reflections for refinement. *R*-factors based on *F*^2^ are statistically about twice as large as those based on *F*, and *R*- factors based on ALL data will be even larger.

### Fractional atomic coordinates and isotropic or equivalent isotropic displacement parameters (Å^2^)

**Table:**

	*x*	*y*	*z*	*U*_iso_*/*U*_eq_	Occ. (<1)
Mo1	0.22547 (6)	0.43949 (6)	0.09513 (2)	0.00816 (11)	
Mo2	0.71279 (6)	0.32538 (6)	0.28488 (2)	0.00936 (11)	
Mo3	0.77392 (6)	0.80749 (6)	0.52742 (2)	0.00941 (11)	
Mo4	0.74619 (6)	0.04587 (6)	0.08587 (2)	0.01000 (11)	
Mo5	0.18593 (7)	0.71922 (7)	0.30929 (3)	0.01203 (11)	
Ag1	0.6084 (2)	0.8428 (4)	0.3355 (3)	0.0375 (8)	0.486 (11)
Ag2	0.6351 (7)	0.8417 (3)	0.2935 (6)	0.056 (2)	0.408 (11)
Co1	0.66729 (10)	0.58420 (10)	0.11583 (4)	0.0087 (2)	0.920 (6)
Al1	0.66729 (10)	0.58420 (10)	0.11583 (4)	0.0087 (2)	0.080 (6)
Co2	0.68200 (11)	0.32794 (11)	0.49318 (4)	0.0083 (3)	0.742 (6)
Al2	0.68200 (11)	0.32794 (11)	0.49318 (4)	0.0083 (3)	0.258 (6)
Co3	0.27664 (11)	0.92534 (11)	0.12614 (4)	0.0076 (3)	0.737 (6)
Al3	0.27664 (11)	0.92534 (11)	0.12614 (4)	0.0076 (3)	0.263 (6)
Co4	0.24922 (13)	0.19504 (13)	0.26423 (5)	0.0088 (3)	0.542 (6)
Al4	0.24922 (13)	0.19504 (13)	0.26423 (5)	0.0088 (3)	0.458 (6)
O1	0.6232 (6)	0.6325 (6)	0.5034 (2)	0.0151 (8)	
O2	0.2678 (6)	0.9019 (6)	0.2444 (2)	0.0170 (8)	
O3	0.3541 (5)	0.6292 (6)	0.1188 (2)	0.0141 (7)	
O4	0.6898 (6)	0.3752 (6)	0.3811 (2)	0.0201 (8)	
O5	0.6529 (6)	0.5519 (6)	0.2336 (2)	0.0163 (8)	
O6	0.9815 (6)	0.9588 (6)	0.1222 (2)	0.0216 (9)	
O7	0.2186 (7)	0.4863 (6)	0.2692 (2)	0.0260 (9)	
O8	0.3258 (6)	0.7020 (6)	0.3915 (2)	0.0201 (8)	
O9	1.0223 (6)	0.7103 (6)	0.5056 (2)	0.0203 (8)	
O10	0.2933 (6)	0.3839 (6)	0.0016 (2)	0.0198 (8)	
O11	0.7036 (6)	0.0327 (6)	0.4785 (2)	0.0188 (8)	
O12	0.5836 (5)	0.8879 (5)	0.1247 (2)	0.0135 (7)	
O13	0.2675 (5)	0.2115 (5)	0.1509 (2)	0.0132 (7)	
O14	−0.0592 (6)	0.7932 (7)	0.3351 (3)	0.0304 (10)	
O15	0.9585 (6)	0.2166 (6)	0.2630 (2)	0.0197 (8)	
O16	−0.0250 (6)	0.5351 (6)	0.1053 (2)	0.0209 (9)	
O17	0.7454 (6)	0.0344 (6)	−0.0127 (2)	0.0222 (9)	
O18	0.7497 (7)	0.8501 (6)	0.6254 (2)	0.0226 (9)	
O19	0.5474 (6)	0.1619 (6)	0.2671 (2)	0.0195 (8)	
O20	0.6641 (7)	0.2928 (6)	0.1097 (2)	0.0236 (9)	

### Atomic displacement parameters (Å^2^)

**Table:**

	*U*^11^	*U*^22^	*U*^33^	*U*^12^	*U*^13^	*U*^23^
Mo1	0.0090 (2)	0.0058 (2)	0.0104 (2)	−0.00328 (15)	−0.00181 (15)	0.00175 (15)
Mo2	0.0101 (2)	0.0082 (2)	0.0097 (2)	−0.00186 (16)	−0.00103 (15)	0.00114 (15)
Mo3	0.0103 (2)	0.0078 (2)	0.0111 (2)	−0.00401 (16)	−0.00117 (15)	0.00059 (15)
Mo4	0.0115 (2)	0.0074 (2)	0.0121 (2)	−0.00496 (16)	0.00168 (16)	0.00012 (15)
Mo5	0.0115 (2)	0.0104 (2)	0.0147 (2)	−0.00390 (17)	−0.00107 (16)	0.00338 (16)
Ag1	0.0138 (6)	0.0340 (8)	0.065 (2)	−0.0072 (5)	−0.0068 (7)	0.0149 (8)
Ag2	0.0357 (14)	0.0136 (8)	0.120 (5)	0.0020 (7)	−0.043 (2)	−0.0071 (13)
Co1	0.0084 (4)	0.0066 (4)	0.0113 (4)	−0.0023 (3)	−0.0010 (3)	0.0013 (3)
Al1	0.0084 (4)	0.0066 (4)	0.0113 (4)	−0.0023 (3)	−0.0010 (3)	0.0013 (3)
Co2	0.0082 (4)	0.0073 (4)	0.0095 (4)	−0.0021 (3)	−0.0013 (3)	0.0007 (3)
Al2	0.0082 (4)	0.0073 (4)	0.0095 (4)	−0.0021 (3)	−0.0013 (3)	0.0007 (3)
Co3	0.0083 (4)	0.0054 (4)	0.0093 (4)	−0.0019 (3)	−0.0014 (3)	0.0003 (3)
Al3	0.0083 (4)	0.0054 (4)	0.0093 (4)	−0.0019 (3)	−0.0014 (3)	0.0003 (3)
Co4	0.0100 (5)	0.0079 (5)	0.0088 (5)	−0.0028 (3)	−0.0012 (3)	0.0007 (3)
Al4	0.0100 (5)	0.0079 (5)	0.0088 (5)	−0.0028 (3)	−0.0012 (3)	0.0007 (3)
O1	0.0148 (18)	0.0153 (19)	0.0155 (18)	−0.0040 (15)	−0.0011 (14)	0.0011 (14)
O2	0.0164 (19)	0.0146 (19)	0.0187 (19)	−0.0014 (15)	0.0047 (15)	0.0024 (15)
O3	0.0118 (17)	0.0158 (19)	0.0168 (18)	−0.0074 (15)	−0.0017 (14)	−0.0006 (14)
O4	0.025 (2)	0.021 (2)	0.0146 (19)	−0.0043 (17)	−0.0020 (16)	0.0007 (16)
O5	0.020 (2)	0.0146 (19)	0.0154 (18)	−0.0041 (16)	−0.0071 (15)	0.0034 (15)
O6	0.0147 (19)	0.024 (2)	0.028 (2)	−0.0076 (17)	−0.0049 (16)	0.0013 (17)
O7	0.034 (2)	0.023 (2)	0.024 (2)	−0.0126 (19)	0.0037 (18)	−0.0011 (17)
O8	0.021 (2)	0.018 (2)	0.021 (2)	−0.0043 (17)	−0.0042 (16)	0.0013 (16)
O9	0.0133 (18)	0.021 (2)	0.027 (2)	−0.0051 (16)	0.0014 (16)	−0.0007 (17)
O10	0.028 (2)	0.017 (2)	0.0134 (18)	−0.0040 (17)	−0.0023 (16)	0.0000 (15)
O11	0.022 (2)	0.0139 (19)	0.021 (2)	−0.0055 (16)	−0.0011 (16)	0.0030 (15)
O12	0.0145 (18)	0.0065 (17)	0.0194 (19)	−0.0021 (14)	0.0005 (14)	−0.0014 (14)
O13	0.0117 (17)	0.0104 (17)	0.0167 (18)	−0.0003 (14)	−0.0027 (14)	0.0028 (14)
O14	0.016 (2)	0.036 (3)	0.039 (3)	−0.0045 (19)	0.0043 (18)	0.003 (2)
O15	0.017 (2)	0.016 (2)	0.025 (2)	0.0008 (16)	0.0014 (16)	−0.0003 (16)
O16	0.0109 (18)	0.018 (2)	0.033 (2)	−0.0019 (16)	−0.0022 (16)	0.0047 (17)
O17	0.032 (2)	0.021 (2)	0.0155 (19)	−0.0117 (18)	0.0034 (17)	0.0006 (16)
O18	0.036 (2)	0.018 (2)	0.0149 (19)	−0.0101 (18)	0.0015 (17)	−0.0017 (16)
O19	0.023 (2)	0.016 (2)	0.021 (2)	−0.0087 (17)	−0.0081 (16)	0.0035 (15)
O20	0.031 (2)	0.0101 (19)	0.031 (2)	−0.0083 (17)	0.0058 (18)	−0.0035 (16)

### Geometric parameters (Å, º)

**Table:**

Mo1—O16	1.720 (4)	Ag2—O5	2.284 (5)
Mo1—O10	1.726 (4)	Co1—O20	2.033 (4)
Mo1—O3	1.791 (4)	Co1—O16^iii^	2.072 (4)
Mo1—O13	1.813 (4)	Co1—O5	2.076 (4)
Mo2—O4	1.735 (4)	Co1—O10^iv^	2.082 (4)
Mo2—O15	1.740 (4)	Co1—O12	2.084 (4)
Mo2—O5	1.771 (4)	Co1—O3	2.107 (4)
Mo2—O19	1.796 (4)	Co2—O4	1.987 (4)
Mo3—O9	1.736 (4)	Co2—O9^v^	1.994 (4)
Mo3—O11^i^	1.751 (4)	Co2—O8^vi^	2.033 (4)
Mo3—O18	1.754 (4)	Co2—O11	2.050 (4)
Mo3—O1	1.812 (4)	Co2—O1^vi^	2.055 (4)
Mo4—O17	1.739 (4)	Co2—O1	2.087 (4)
Mo4—O6^ii^	1.750 (4)	Co3—O6^vii^	1.995 (4)
Mo4—O20	1.757 (4)	Co3—O17^iv^	2.014 (4)
Mo4—O12^ii^	1.807 (4)	Co3—O3	2.028 (4)
Mo5—O14	1.705 (4)	Co3—O13^i^	2.037 (4)
Mo5—O7	1.757 (4)	Co3—O12	2.068 (4)
Mo5—O8	1.758 (4)	Co3—O2	2.080 (4)
Mo5—O2	1.828 (4)	Co4—O18^vi^	1.957 (4)
Ag1—O14^iii^	2.237 (5)	Co4—O15^vii^	1.970 (4)
Ag1—O19^i^	2.455 (4)	Co4—O13	1.994 (4)
Ag1—O8	2.484 (4)	Co4—O7	1.996 (4)
Ag2—O14^iii^	2.210 (5)	Co4—O19	2.015 (4)
Ag2—O19^i^	2.225 (5)	Co4—O2^ii^	2.056 (4)
			
O16—Mo1—O10	111.2 (2)	O5—Co1—O3	87.85 (15)
O16—Mo1—O3	106.92 (18)	O10^iv^—Co1—O3	96.81 (15)
O10—Mo1—O3	106.34 (18)	O12—Co1—O3	77.15 (15)
O16—Mo1—O13	106.10 (18)	O4—Co2—O9^v^	90.75 (17)
O10—Mo1—O13	107.77 (18)	O4—Co2—O8^vi^	176.36 (17)
O3—Mo1—O13	118.55 (17)	O9^v^—Co2—O8^vi^	88.98 (17)
O4—Mo2—O15	108.65 (19)	O4—Co2—O11	90.57 (16)
O4—Mo2—O5	107.77 (18)	O9^v^—Co2—O11	89.95 (17)
O15—Mo2—O5	109.23 (18)	O8^vi^—Co2—O11	93.06 (16)
O4—Mo2—O19	107.54 (19)	O4—Co2—O1^vi^	91.49 (16)
O15—Mo2—O19	110.94 (19)	O9^v^—Co2—O1^vi^	177.73 (16)
O5—Mo2—O19	112.58 (18)	O8^vi^—Co2—O1^vi^	88.76 (16)
O9—Mo3—O11^i^	109.69 (19)	O11—Co2—O1^vi^	90.41 (16)
O9—Mo3—O18	107.9 (2)	O4—Co2—O1	87.19 (16)
O11^i^—Mo3—O18	108.25 (19)	O9^v^—Co2—O1	97.01 (16)
O9—Mo3—O1	109.13 (18)	O8^vi^—Co2—O1	89.24 (15)
O11^i^—Mo3—O1	111.73 (18)	O11—Co2—O1	172.71 (16)
O18—Mo3—O1	110.08 (18)	O1^vi^—Co2—O1	82.72 (16)
O17—Mo4—O6^ii^	112.9 (2)	O6^vii^—Co3—O17^iv^	82.08 (17)
O17—Mo4—O20	107.5 (2)	O6^vii^—Co3—O3	99.95 (16)
O6^ii^—Mo4—O20	109.6 (2)	O17^iv^—Co3—O3	92.94 (16)
O17—Mo4—O12^ii^	107.04 (18)	O6^vii^—Co3—O13^i^	93.72 (16)
O6^ii^—Mo4—O12^ii^	107.18 (18)	O17^iv^—Co3—O13^i^	97.00 (16)
O20—Mo4—O12^ii^	112.78 (18)	O3—Co3—O13^i^	164.09 (15)
O14—Mo5—O7	108.7 (2)	O6^vii^—Co3—O12	177.23 (16)
O14—Mo5—O8	108.4 (2)	O17^iv^—Co3—O12	95.29 (16)
O7—Mo5—O8	109.1 (2)	O3—Co3—O12	79.28 (14)
O14—Mo5—O2	110.1 (2)	O13^i^—Co3—O12	87.43 (14)
O7—Mo5—O2	112.59 (18)	O6^vii^—Co3—O2	92.31 (16)
O8—Mo5—O2	107.84 (18)	O17^iv^—Co3—O2	173.71 (17)
O14^iii^—Ag1—O19^i^	98.42 (17)	O3—Co3—O2	90.81 (15)
O14^iii^—Ag1—O8	140.69 (19)	O13^i^—Co3—O2	80.47 (15)
O19^i^—Ag1—O8	120.42 (15)	O12—Co3—O2	90.37 (15)
O14^iii^—Ag2—O19^i^	106.6 (2)	O18^vi^—Co4—O15^vii^	92.13 (18)
O14^iii^—Ag2—O5	99.1 (2)	O18^vi^—Co4—O13	173.62 (17)
O19^i^—Ag2—O5	138.3 (5)	O15^vii^—Co4—O13	90.93 (16)
O20—Co1—O16^iii^	92.15 (17)	O18^vi^—Co4—O7	94.80 (17)
O20—Co1—O5	88.35 (16)	O15^vii^—Co4—O7	91.14 (18)
O16^iii^—Co1—O5	95.76 (16)	O13—Co4—O7	90.73 (16)
O20—Co1—O10^iv^	92.55 (16)	O18^vi^—Co4—O19	86.79 (17)
O16^iii^—Co1—O10^iv^	79.59 (16)	O15^vii^—Co4—O19	177.71 (17)
O5—Co1—O10^iv^	175.29 (16)	O13—Co4—O19	89.94 (15)
O20—Co1—O12	163.71 (16)	O7—Co4—O19	90.96 (18)
O16^iii^—Co1—O12	104.13 (16)	O18^vi^—Co4—O2^ii^	92.52 (16)
O5—Co1—O12	90.12 (15)	O15^vii^—Co4—O2^ii^	86.18 (16)
O10^iv^—Co1—O12	90.31 (15)	O13—Co4—O2^ii^	82.09 (15)
O20—Co1—O3	86.59 (16)	O7—Co4—O2^ii^	172.29 (17)
O16^iii^—Co1—O3	176.14 (16)	O19—Co4—O2^ii^	91.85 (16)

Symmetry codes: (i) *x*, *y*+1, *z*; (ii) *x*, *y*−1, *z*; (iii) *x*+1, *y*, *z*; (iv) −*x*+1, −*y*+1, −*z*; (v) −*x*+2, −*y*+1, −*z*+1; (vi) −*x*+1, −*y*+1, −*z*+1; (vii) *x*−1, *y*, *z*.

## References

[bb1] Brandenburg, K. & Putz, H. (2001). *DIAMOND*. Crystal Impact GbR, Bonn, Allemagne.

[bb2] Brik, M. G. & Avram, C. N. (2011). *J. Lumin.* **131**, 2642–2645.

[bb3] Brown, I. D. & Altermatt, D. (1985). *Acta Cryst.* B**41**, 244–247.

[bb4] Duisenberg, A. J. M. (1992). *J. Appl. Cryst.* **25**, 92–96.

[bb5] Engel, J. M., Ahsbahs, H., Fuess, H. & Ehrenberg, H. (2009). *Acta Cryst.* B**65**, 29–35. 10.1107/S0108768108038536 19155556

[bb6] Ennajeh, I., Zid, M. F. & Driss, A. (2013). *Acta Cryst.* E**69**, i54–i55. 10.1107/S1600536813022046 PMC3884375 24426975

[bb7] Farrugia, L. J. (2012). *J. Appl. Cryst.* **45**, 849–854.

[bb8] Feng, P., Bu, X. & Stucky, G. D. (1997). *J. Solid State Chem.* **129**, 328–333.

[bb9] Harms, K. & Wocadlo, S. (1995). *XCAD4*. Université de Marburg, Allemagne.

[bb10] Hermanowicz, K., Mączka, M., Wołcyrz, M., Tomaszewski, P. E., Paściak, M. & Hanuza, J. (2006). *J. Solid State Chem.* **179**, 685–695.

[bb11] Huyghe, M., Lee, M.-R., Quarton, M. & Robert, F. (1991). *Acta Cryst.* C**47**, 244–246.

[bb12] Ivanov, K., Krustev, S. & Litcheva, P. (1998). *J. Alloys Compd*, **279**, 132–135.

[bb13] Judeinstein, P., Titman, J., Stamm, M. & Schmidt, H. (1994). *Chem. Mater.* **6**, 127–134.

[bb14] Lii, K.-H. & Ye, J. (1997). *J. Solid State Chem.* **131**, 131–137.

[bb15] Macíček, J. & Yordanov, A. (1992). *J. Appl. Cryst.* **25**, 73–80.

[bb16] Méndez-Blas, A., Rico, M., Volkov, V., Cascales, C., Zaldo, C., Coya, C., Kling, A. & Alves, L. C. (2004). *J. Phys. Condens. Matter*, **16**, 2139–2160.

[bb17] Muessig, E., Bramnik, K. G. & Ehrenberg, H. (2003). *Acta Cryst.* B**59**, 611–616. 10.1107/s0108768103016598 14586081

[bb18] North, A. C. T., Phillips, D. C. & Mathews, F. S. (1968). *Acta Cryst.* A**24**, 351–359.

[bb19] Sanz, F., Parada, C., Rojo, J. M., Ruiz-Valero, C. & Saez-Puche, R. (1999). *J. Solid State Chem.* **145**, 604–611.

[bb20] Sheldrick, G. M. (2008). *Acta Cryst.* A**64**, 112–122. 10.1107/S0108767307043930 18156677

[bb21] Tsyrenova, G. D., Solodovnikov, S. F., Khaikina, E. G., Khobrakova, E. T., Bazarova, Zh. G. & Solodovnikova, Z. A. (2004). *J. Solid State Chem.* **177**, 2158–2167.

